# A robust small-object detection model for UAV aerial imagery under complex background clutter

**DOI:** 10.1371/journal.pone.0352244

**Published:** 2026-08-04

**Authors:** Yong He, Renfeng Xiao, Yifan Tang, Yufan Pang

**Affiliations:** College of Artificial Intelligence, Changsha University of Science and Technology, Changsha, China; Communication University of Zhejiang, CHINA

## Abstract

To tackle the challenge of detecting small targets in UAV imagery, this paper proposes PF-DETR, an enhanced object detection model based on RT-DETR, designed to improve detection accuracy in complex scenes. The improvements are primarily reflected in the following aspects. First, a P2 detection head is added to extend the feature pyramid to finer scales, thereby enhancing the ability of shallow features to detect small targets. Second, a Pyramidal Hierarchical Frequency-Domain Fusion (PHF) module is introduced. By combining wavelet pooling with high- and low-frequency attention, the module effectively extracts and fuses multi-scale features, reduces information loss, and improves detection accuracy for small targets. Finally, the backbone network is restructured through the design of a lightweight BasicBlock_FasterNet_Rep module, which integrates FasterNet and RepVGG-style re-parameterization. This restructuring significantly reduces model complexity and parameters while strengthening multi-scale feature extraction. Experimental results on the VisDrone2019 dataset show that the improved PF-DETR achieves a notable performance boost: compared to the original model, mAP@0.5 increases by 5.4%, while the number of parameters is reduced by 25.0%. The computational cost increases by about 35.0%, but this comes with higher accuracy, resulting in a favorable balance between detection performance and model efficiency. Overall, these improvements enhance the model’s robustness and accuracy in detecting multi-scale and small targets in complex and cluttered scenes.

## Introduction

With the rapid development of unmanned aerial vehicle (UAV) technology, UAV systems equipped with visual sensors and artificial intelligence algorithms have been widely used in urban management, traffic monitoring, agricultural monitoring, emergency rescue [1], and other fields. UAV aerial images are characterized by large variations in viewpoint, complex and diverse backgrounds, and significant differences in target scale. In particular, small targets in images (e.g., pedestrians and vehicles) are difficult to detect due to their low resolution and weak feature representations [2], which has become a major challenge in object detection research. Although traditional object detection methods, such as YOLO, R-CNN [3], and Faster R-CNN [4], have achieved good performance in some scenarios, they still suffer from insufficient accuracy and high miss rates for small objects [5] in UAV aerial images, especially when handling multi-scale objects and complex backgrounds. This remains a common challenge in small-object detection research. Currently, many researchers in the field of UAV object detection are improving YOLO-series algorithms to address the difficulties of detecting objects in aerial images and to enhance detection accuracy. For example, Zhu et al. [6] proposed the TPH-YOLOv5 algorithm, which adds a small-object detection head and a Transformer prediction head (TPH), and incorporates a convolutional block attention module (CBAM) to improve object localization accuracy in high-density scenes. Similarly, Lin et al. [7] proposed the HTH-YOLOv5 algorithm, which significantly improves the detection accuracy of small objects in UAV images by introducing a Hybrid Transformer detection head (HTH) alongside CBAM.

In recent years, the DETR algorithm [8], based on the Transformer architecture, has gained popularity in object detection due to the advantages of its attention mechanism in capturing semantic information. However, the DETR model still faces challenges, including high complexity, difficulty in achieving training convergence, and unsatisfactory performance in small-object detection [9]. To address these issues, Zhu et al. [10] proposed Deformable DETR, which utilizes a deformable attention mechanism to extract multi-scale features, thereby reducing model complexity and accelerating convergence. Zhao et al. [11] introduced RT-DETR, which significantly enhances detection accuracy and real-time performance through an efficient hybrid encoder and IoU-aware query selection, laying a theoretical foundation for applying DETR models to real-time object detection in UAV applications.

Subsequently, researchers have made several improvements to RT-DETR: Zhou et al. [12] proposed the UAV-DETR algorithm, which effectively enhances the detection accuracy of small objects in UAV images by introducing a Channel-Aware Module (CAS), a Scale-Optimized Enhanced Pyramid (SOEP) module, and a Contextual Spatial Alignment Module (CSAM). However, this method still faces limitations when processing low-resolution images. Su et al. [13] introduced the DRT-DETR algorithm, which significantly improves detection accuracy and computational efficiency by incorporating a Fast Multi-scale Attention Feature Extraction Module (Faster-EMA) and a Weighted Bidirectional Cross-scale Feature Fusion Module (Bi-CCFM). Nevertheless, this approach still carries the risk of missed detections when handling small targets in complex backgrounds. Shen et al. [14] proposed the TinyDef-DETR algorithm, which enhances the model’s ability to detect small objects in power line defect inspection by introducing a lossless downsampling module, edge-enhanced convolution, a cross-stage dual-domain multi-scale attention module, and a focus-aware regression loss function. However, the model is relatively sensitive to background noise and may exhibit false detections or missed detections in natural environments or cluttered scenes. Tong et al. [15] proposed the ACD-DETR algorithm, which improves small-object detection performance by incorporating a Multi-scale Edge Enhancement Fusion Module (MSEFM), a Global Boundary Calibration module (OG-BC), and a Dynamic Position Bias Attention module (DPB-AIFI). Despite these improvements, the model’s stability and accuracy still require further enhancement when handling complex background scenarios.

In summary, several challenges persist in complex scenarios such as drone aerial imagery, including insufficient feature extraction by the backbone network [16], high computational complexity, feature loss due to traditional pooling operations [17], and low localization accuracy for small objects. These issues hinder the practical effectiveness of existing methods in complex drone application scenarios.

To achieve a better balance between speed and accuracy in object detection while addressing the above issues and maintaining the end-to-end real-time detection paradigm of RT-DETR, this study adopts a coordinated design of shallow-detail preservation, frequency-aware feature interaction, and lightweight efficient reconstruction to enhance the detectability and robustness of small objects in complex UAV scenes. Based on RT-DETR, we develop an improved framework termed PF-DETR. The main contributions are as follows:

(1) To improve the detection of tiny objects in aerial imagery, we incorporate an additional P2 detection head into the feature pyramid of RT-DETR, following prior small-object detection studies that enhance tiny-object perception by introducing finer-scale prediction layers or additional small-object detection heads [6]- [7]. In aerial scenes, targets often exhibit substantial scale variation and dense occlusion. By extending the feature pyramid to a finer-resolution stage, the proposed design allows shallow high-resolution features to contribute more directly to localization and recall, thereby improving detection performance for small targets.
(2) To alleviate detail loss during multi-scale feature aggregation, we develop a Pyramid Hierarchical Frequency Fusion (PHF) design by integrating wavelet pooling/unpooling [18] and HiLo attention [19] into the RT-DETR neck. Specifically, wavelet-based decomposition is used to preserve high-frequency details and low-frequency structure during downsampling and upsampling, while HiLo attention is incorporated into the AIFI interaction process to better balance local detail modeling and global context aggregation with relatively low computational cost. The contribution here lies not in introducing entirely new frequency-domain or attention operators, but in their coordinated integration for frequency-aware feature fusion in UAV small-object detection.
(3) To improve deployment efficiency while maintaining feature extraction capability, we construct a lightweight backbone block for RT-DETR by combining FasterNet partial convolution [20] with RepVGG-style structural re-parameterization [21]. This design adapts existing efficient architectural components to the UAV small-object detection setting, reducing parameter count and inference cost while preserving multi-scale representation ability. Thus, the main contribution is reflected in the lightweight adaptation and system-level integration of these techniques within PF-DETR.

It should be noted that the key technical components adopted in PF-DETR, including the finer-scale prediction strategy implemented by the P2 detection head, wavelet-based pooling/unpooling, HiLo attention, FasterNet, and RepVGG-style structural re-parameterization, are derived from existing studies or established design practices in object detection. Therefore, the contribution of this work does not lie in proposing entirely new basic modules, but in the task-oriented integration and adaptation of these techniques within the RT-DETR framework for UAV small-object detection under complex background clutter.

Experimental results demonstrate that PF-DETR achieves a significant performance boost on the VisDrone2019 dataset [22]. Compared to the original RT-DETR-R18 model, the improved model achieves an mAP@0.5 of 0.525, representing a 5.4% improvement, while reducing the number of parameters by 25.0%. Despite the increase in computational cost by 35.0%, the model maintains practicality and achieves accuracy gains, demonstrating a co-optimization of accuracy and lightweight design. These improvements effectively enhance the model’s robustness and accuracy in complex scenarios, as well as in multi-scale and small-object detection.

## Related work

To better situate our study and clarify its positioning, we briefly review related research along three directions: multi-scale detail preservation, Transformer-based real-time detection, and frequency-aware enhancement.

UAV small-object detection is often improved by strengthening multi-scale representations and preserving high-resolution details, since repeated downsampling can readily erase the already-weak visual cues of tiny targets in cluttered backgrounds. A common practice is to densify the feature pyramid or introduce finer-scale prediction layers, allowing shallow features to contribute more directly to localization and recall. While effective for scale-imbalanced aerial scenes, these designs can still struggle when background textures dominate the signal.

A second research direction focuses on global context modelling and cross-scale feature interaction. Transformer-based detectors (including DETR-style frameworks) leverage attention to capture long-range dependencies, and real-time variants such as RT-DETR streamline the encoder–decoder pipeline to improve efficiency while retaining end-to-end training. Follow-up RT-DETR adaptations for aerial imagery typically augment context-aware or cross-scale fusion components; however, reliably detecting small objects under heavy clutter remains difficult due to limited fine-grained evidence and information loss during pyramid aggregation.

Complementary to spatial-domain fusion, frequency-aware enhancement has been explored to mitigate detail attenuation. Wavelet-based decomposition, in particular, can decompose features into low- and high-frequency components, helping retain edge and texture cues that are critical for tiny targets. Against this background, PF-DETR is positioned as a coordinated design that: (i) brings shallow, high-resolution signals closer to the prediction stage through an earlier-scale output; (ii) strengthens detail–context complementarity under clutter via multi-resolution frequency decomposition and hierarchical fusion; and (iii) maintains real-time deployability with a compact backbone design that is amenable to efficient inference.

## RT-DETR overview

RT-DETR (Real-Time Detection Transformer) is an object detection model based on the Transformer architecture, designed for efficient, real-time object detection. The model uses ResNet18 [23] as the backbone network to extract multi-scale features from the input image. RT-DETR achieves efficient feature fusion by integrating the AIFI (Intra-scale Feature Interaction) and CCFM (Cross-scale Feature Fusion) modules. The AIFI module employs a multi-head attention mechanism to facilitate interactions among high-level features, thereby enhancing feature representation within the same scale. Meanwhile, the CCFM module uses RepC3 to fuse features from different scales, improving detection capability for multi-scale objects. The decoder optimizes object position prediction with an IoU-aware query selection mechanism and accelerates model convergence through contrastive denoising training [24,25], improving training efficiency. Ultimately, RT-DETR uses the Hungarian matching algorithm to match predicted bounding boxes with ground truth boxes, ensuring precision and accuracy in object detection. Unlike traditional object detection methods, RT-DETR eliminates the anchor mechanism and Non-Maximum Suppression (NMS), resulting in a more streamlined and efficient end-to-end training process, making it ideal for application scenarios that require fast inference. Through these innovations, RT-DETR significantly enhances both the accuracy and speed of real-time object detection, particularly excelling in detecting multi-scale and small objects in complex environments.

## PF-DETR model

**Fig 1:**
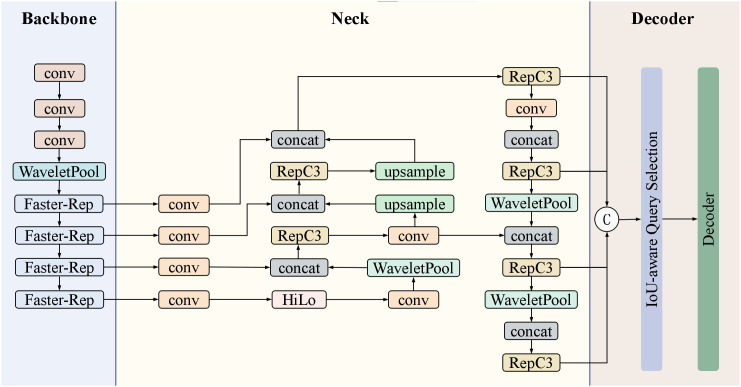
PF-DETR network structure.

Based on RT-DETR, improvements have been made to the backbone network, neck network, and feature fusion methods. The architecture of the improved PF-DETR network is illustrated in Fig 1. To create a lightweight and efficient backbone network, the FasterNet module (BasicBlock_FasterNet_Rep), integrated with RepVGG’s re-parameterization technique, is used to reduce the number of parameters. To enhance multi-scale feature fusion, a frequency-domain perceptual fusion module (PHFusion) based on WaveletPooling/UnPooling and HiLo attention is introduced. This module reduces information loss and improves feature discrimination. Additionally, a denser feature pyramid is constructed by extending the P2 feature layer, improving the model’s ability to perceive and precisely position small targets.

### Lightweight feature extraction network: FasterNet_Rep backbone network

FasterNet effectively reduces computational complexity and improves target detection accuracy by optimizing FLOPs. Designed by Chen et al. at CVPR 2023, FasterNet is a lightweight neural network. The basic structure of the FasterNet module consists of PConv and pointwise convolution (PWConv). This network demonstrates faster detection speed and lower parameter and computational complexity across various devices. To further enhance the model’s performance, the RepConv module, featuring a multi-branch architecture, replaces the original PConv module. During training, RepConv is decomposed into multiple convolution-normalization layers, allowing the network to learn richer feature-mapping relationships and facilitating optimization. During inference, the RepConv block is re-parameterized into a single standard convolution layer, reducing computational steps and memory usage, thus accelerating inference. Specifically, the module extracts multi-scale features through parallel branches, increasing feature diversity and providing more comprehensive spatial information. This improves the model’s feature representation capacity and generalization performance. The RepConv module enables efficient and lightweight detection on UAV platforms. By using structural re-parameterization technology, the RepConv module converts the multi-branch structure into an equivalent single-branch convolution operation. This conversion relies on techniques like zero-filling and convolution kernel fusion, ultimately simplifying the complex multi-branch structure into a standard 3 × 3 convolution. As a result, no additional computational overhead is introduced during inference, ensuring the model’s practical efficiency. Overall, the FasterNet_Rep backbone reduces computational complexity while improving detection accuracy through multi-scale feature fusion during training and efficient structural re-parameterization during inference.

**Fig 2:**
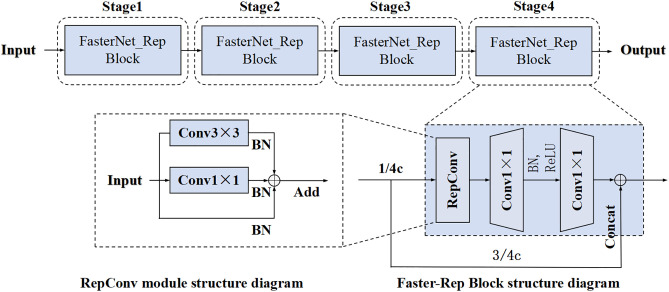
FasterNet_Rep backbone network.

As shown in Fig 2, the proposed FasterNet_Rep backbone adopts a lightweight re-parameterized design to enhance feature extraction while reducing computational complexity.

### Addition of a small-object detection head (P2)

Small objects are challenging to detect using the standard P3, P4, and P5 detection layers. To address this, we introduce an additional P2 detection head on top of the conventional R18 model. This head operates on intermediate-layer features, enhancing the model’s ability to detect small targets across scales. By incorporating the P2 head, the network can leverage higher-resolution feature maps, improving its robustness to objects of varying sizes.

### PHF module

In the neck feature pyramid of PF-DETR, we introduce a Pyramid Hierarchical Frequency Fusion (PHF) module by coupling AIFI-HiLo with WaveletPool/UnPool. Much like observing a landscape from varying altitudes, UAV aerial imagery presents objects whose salient details fade as perspective shifts across scales. PHF is designed to counteract this attenuation by embracing the intrinsic multi-resolution nature of visual signals. Through wavelet transformation, features are explicitly decomposed into complementary high- and low-frequency components, analogous to separating the fine ripples from the underlying terrain. HiLo attention is then employed to hierarchically model and reunite these frequency bands, allowing discriminative details and global structure to co-evolve. This harmonious fusion strengthens multi-scale representations and significantly enhances the detectability of small aerial targets.

WaveletPool: Conventional downsampling often resembles erosion, gradually washing away the delicate contours of tiny objects. WaveletPool, in contrast, performs an explicit sub-band decomposition that carefully preserves both structure and detail while reducing spatial resolution. By reorganizing frequency-specific information into the channel dimension, it retains high-frequency cues such as edges and local textures—signals that are vital for distinguishing small targets against cluttered backgrounds. Compared with max or average pooling, WaveletPool suppresses redundant background responses and delivers a more expressive, information-rich representation to subsequent attention-based interactions.

WaveletUnPool: Mirroring the decomposition process, WaveletUnPool acts as a restorative mechanism during upsampling. Through wavelet synthesis, it reconstructs spatial resolution by reintegrating previously compressed detail components, much like reassembling scattered fragments into a coherent whole. This principled inverse transform mitigates the blurring and localization ambiguity commonly introduced by simple interpolation, providing stronger spatial support for cross-scale feature fusion and enabling more precise localization of small aerial objects.

AIFI-HiLo: Operating on frequency-decomposed features, AIFI-HiLo adopts a dual-branch attention strategy that reflects the balance between detail and context in natural perception. The high-frequency branch focuses on local, fine-grained patterns, while the low-frequency branch captures global structure and long-range dependencies. Their hierarchical interaction and final fusion reinforce both textural sharpness and structural coherence along the multi-scale pathway, allowing target-specific features to emerge robustly even under complex and cluttered aerial scenes.

#### Wavelet pooling (WaveletPool).

WaveletPool and WaveletUnPool are wavelet-transform-based pooling and unpooling methods designed to enhance the model’s small-target detection performance in UAV aerial imagery by effectively extracting multi-scale image features. The WaveletPool operation applies four Haar wavelet filters (low-pass, horizontal high-pass, vertical high-pass, and diagonal high-pass) during pooling to capture both low- and high-frequency image features, thereby effectively extracting subtle information.

**Wavelet Basis Selection**. In this work, we adopt the Haar wavelet for WaveletPool and WaveletUnPool. This choice is motivated by three key considerations that are crucial for UAV small-object detection:

(1) Edge/detail sensitivity: The Haar wavelet preserves high-frequency components, such as edges and local textures, which are critical for tiny-object representation in cluttered scenes.
(2) Computational efficiency: Its short support and parameter-free filters enable efficient implementation with minimal overhead.
(3) Stable reconstruction: Its analysis/synthesis pair supports more faithful spatial reconstruction than interpolation-based upsampling during multi-scale fusion.

Compared with classical frequency-domain methods such as FFT and DCT, wavelet transform is better suited to the PHF design in this work. FFT mainly models global frequency responses and provides limited spatial localization, while DCT focuses more on compact frequency representation. However, UAV small-object detection requires preserving fine-grained local structures under complex background clutter. Wavelet transform provides localized multi-resolution decomposition, which can better retain high-frequency detail cues while maintaining the hierarchical organization of feature maps. Therefore, the advantage of PHF lies not simply in introducing frequency-domain processing, but in enabling hierarchical detail-semantic fusion for small-object detection through wavelet-based decomposition and reconstruction.

Although this study did not include a systematic comparison across different wavelet families (e.g., Daubechies or Symlet), the Haar wavelet was selected for PF-DETR as a practical and efficient choice because of its shortest support, parameter-free fixed filters, and low computational overhead, which are well suited to real-time UAV object detection. Prior studies have shown that wavelet-based pooling/downsampling can better preserve informative structures during resolution reduction than conventional pooling [18]. In addition, Haar-wavelet-based downsampling provides a simple and effective implementation for detail-sensitive visual tasks [26]. Therefore, in the present work, Haar was adopted as an efficient and implementation-friendly wavelet basis for the PHF module.

**CNN Compatibility**. WaveletPool is naturally compatible with convolutional neural networks because its forward process can be implemented as grouped convolutions with fixed kernels and stride 2. In our design, the four sub-band filters (LL, LH, HL, and HH) are replicated along the channel dimension and applied independently to each input channel. Thus, the operation preserves the local receptive-field pattern of CNNs while converting spatial downsampling into frequency-aware feature rearrangement. Unlike conventional max pooling or average pooling, which directly discard part of the spatial information, WaveletPool retains both low-frequency structural information and high-frequency detail cues in different sub-bands, allowing subsequent convolution layers to learn from a richer representation.

For the inverse process, WaveletUnPool is implemented by transposed convolution using the corresponding inverse wavelet filters. This reconstruction operation is also fully compatible with the standard CNN computation graph and enables end-to-end training without modifying the optimization strategy. Since both operations are differentiable and parameter-free, they can be seamlessly inserted into the neck network for feature fusion. In this way, the wavelet-based operators not only preserve the hierarchical inductive bias of CNNs, but also complement CNN feature extraction by explicitly modeling frequency-domain information that is usually entangled in standard convolutional features.

**Boundary Handling and Normalization**. When H or W is not divisible by 2, we use standard padding/cropping consistent with the convolution operator to avoid boundary artefacts. In our experiments, inputs are resized to a fixed resolution (e.g., 640 × 640), so the down/up-sampling operations remain well-defined.

**Fig 3:**
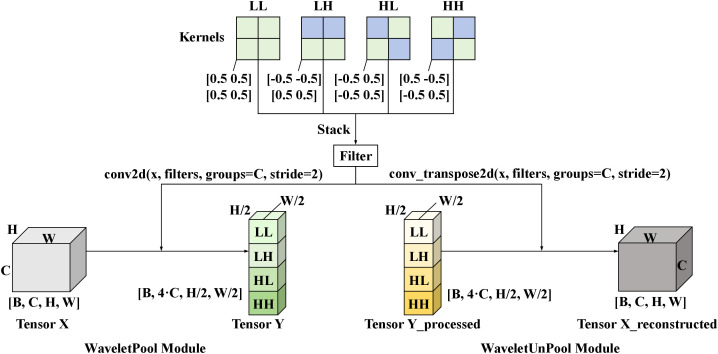
WaveletPool and WaveletUnPool Module Structural Diagram.

As shown in Fig 3, the process of WaveletPool and WaveletUnPool is illustrated, highlighting the operation flow from feature map decomposition into frequency sub-bands (WaveletPool) to the inverse process of feature map reconstruction (WaveletUnPool). The figure demonstrates how multi-scale fusion is achieved by combining low- and high-frequency information, which enhances the detection of small objects under complex backgrounds.

These filters preserve the overall structural characteristics of the image (e.g., terrain or object contours) while retaining fine details of small objects (e.g., edges, textures, and local patterns). Unlike conventional max pooling and average pooling, WaveletPool more effectively exploits the low- and high-frequency information in the image, reducing the feature-map resolution while enhancing the model’s focus on detailed features and preserving more critical information. Spatial information is reorganized and packed into the channel dimension for downsampling, rather than being coarsely discarded. Simultaneously, this operation reduces spatial resolution and computational cost, enabling a more efficient two-dimensional discrete wavelet transform. Equations (1) and (2) describe the forward transformation process of WaveletPool.


$$\begin{array}{c} {\text{W}}_{\varphi }[\text{j}+\text{1},\text{k}]={\text{h}}_{\varphi }[-\text{n}]*{\text{W}}_{\varphi }[\text{j},\text{n}]{|}_{\text{n}=\text{2k},\text{k}\leq \text{0}} \end{array}$$ (1)

$$\begin{array}{c} {\text{W}}_{\psi }[\text{j}+\text{1},\text{k}]={\text{h}}_{\psi }[-\text{n}]*{\text{W}}_{\psi }[\text{j},\text{n}]{|}_{\text{n}=\text{2k},\text{k}\leq \text{0}} \end{array}$$ (2)

Symbol definitions in the equations:Φ and Ψ denote the approximation function and the detail function, respectively. ${\text{W}}_{\phi }$ and ${\text{W}}_{\psi }$ are their respective correlation coefficients. ${\text{h}}_{\phi }\left[-𝔫\right]$ and ${\text{h}}_{\psi }\left[-n\right]$ denote time-domain quantities characterized by scaling and wavelet-direction quantization, where [n] represents the sample time series and [j] denotes the resolution level.

In practical applications, WaveletPool performs convolution using fixed wavelet filter weights, without additional gradient computation or parameter updates. During pooling, each wavelet filter is replicated and stacked to match the number of channels in the input tensor, and the feature-map size is reduced by a convolution operation with a stride of 2. The output feature map has the shape (B, 4*c, h/2, w/2), where B is the batch size, C is the number of input channels, and H and W are the height and width of the input image.

WaveletUnPool serves as an inverse pooling operation to recover the spatial resolution lost during pooling. Using the same wavelet filters as WaveletPool, WaveletUnPool upsamples the feature map via transposed convolution (deconvolution), restoring it to a higher resolution. This process effectively reconstructs the spatial details of small targets while preserving multi-scale features, enabling the model to achieve higher accuracy in small-target detection. After the inverse pooling operation, the shape of the output feature map is restored to (B, C, h, w). Through wavelet synthesis, it recovers the details and structure from the compressed features as much as possible, ensuring high-quality upsampling. Formula (3) describes the unpooling process of the WaveletUnPool module.


$$\begin{array}{c} {\text{W}}_{\varphi }[\text{j},\text{k}]={\text{h}}_{\varphi }[-\text{n}]*{\text{W}}_{\varphi }[\text{j}+\text{1},\text{n}]+{\text{h}}_{\psi }[-\text{n}]*{\text{W}}_{\psi }[\text{j}+\text{1},\text{n}]{|}_{\begin{array}{c} \text{n}=\frac{\text{k}}{\text{2}},\text{k}\leq \text{0} \end{array}} \end{array}$$ (3)

By combining WaveletPool and WaveletUnPool, the network can not only compress information effectively and extract high- and low-frequency features, but also restore image spatial details through the inverse pooling operation when processing UAV aerial images. This improves the model’s performance in small-target detection, particularly for low-contrast and small-size targets, significantly enhancing detection accuracy.

#### AIFI-HiLo module design.

**Fig 4:**
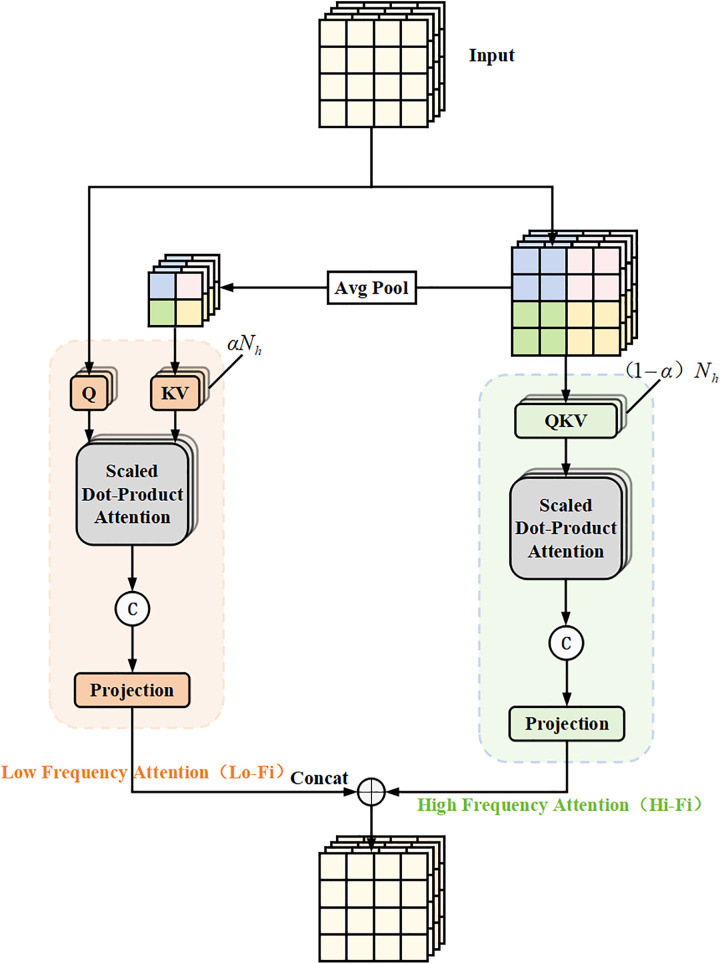
Diagram of HiLo Attention Structure.

The AIFI-HiLo module (a high-/low-frequency attention module) is an AIFI variant constructed by incorporating the HiLo attention mechanism into the original AIFI module. It aims to improve small-target detection by efficiently extracting high- and low-frequency features, particularly for recognizing small targets in complex backgrounds. This module adopts the HiLo attention mechanism to split the multi-head self-attention (MSA) mechanism [27] into high-frequency and low-frequency branches, thereby reducing computational complexity while preserving image details. The specific implementation of HiLo attention is shown in Fig 4, which illustrates the separate processing architecture for high-frequency and low-frequency attention.

Specifically, the workflow of the AIFI-HiLo module is as follows:

High-frequency attention (HI-FI): This branch focuses on fine-grained details in the image, such as edges, textures, and local information of small objects. It models local features using a small attention window, thereby enhancing the model’s response to high-frequency details. HI-FI captures subtle variations in the image, which is especially beneficial for small-target detection. Its computation follows the standard self-attention formulation and can be expressed as:


$$\begin{array}{c} {\text{Attention}}_{\text{Hi}-\text{Fi}}(\text{Q},\text{K},\text{V})=\text{Softmax}(\frac{\text{Q}{\text{K}}^{\text{T}}}{\sqrt{{\text{d}}_{\text{k}}}})\text{V} \end{array}$$ (4)

Low-frequency attention (Lo-Fi): This part mainly focuses on the global structure of the image, such as the contours of large-scale objects and background information. By applying average pooling to window-level features and reducing spatial resolution, Lo-Fi effectively extracts macro-level information from the image while minimizing computational overhead. This process can be expressed as:


$$\begin{array}{c} {\text{Attention}}_{\text{Lo}-\text{Fi}}(\text{Q},\overset{―}{\text{K}},\overset{―}{\text{V}})=\text{Softmax}(\frac{\text{Q}{\overset{―}{\text{K}}}^{\text{T}}}{\sqrt{{\text{d}}_{\text{k}}}})\overset{―}{\text{V}} \end{array}$$ (5)

HiLo’s output is obtained by concatenating the outputs of Hi-Fi and Lo-Fi. The process is shown in Eq. (6), where [...] denotes the concatenation operation.


$$\begin{array}{c} \text{AIFI}-\text{HiLo}(\text{x})=[\text{HiFi}(\text{x}),\text{LoFi}(\text{x})] \end{array}$$ (6)

To further analyze the computational cost of the different branches, the theoretical computational complexity can be expressed as:


$$\begin{array}{c} \text{Hi}-\text{Fi Complexity}=\frac{\text{7}}{\text{4}}ND^{\text{2}}+s^{\text{2}}ND \end{array}$$ (7)

$$\begin{array}{c} \text{Lo}-\text{Fi Complexity}=(\frac{\text{3}}{\text{4}}+\frac{\text{1}}{s^{\text{2}}})ND^{\text{2}}+\frac{\text{1}}{s^{\text{2}}}N^{\text{2}}D \end{array}$$ (8)

Where: N and D denote the number of tokens and the hidden dimension in the attention layer, respectively; s is the window size. This derivation shows that, under high-resolution inputs, by appropriately increasing the window size s, the HiLo module can significantly reduce computational complexity while maintaining feature representation capability, thereby achieving lightweight and efficient performance without compromising the accuracy of small-target detection.

The AIFI HiLo module can simultaneously capture fine-grained details and global context by separately modeling the image’s high-frequency and low-frequency features and then fusing them, thereby significantly improving small-target detection performance.

The PHF module integrates multi-level feature fusion with the separation of high- and low-frequency information, significantly enhancing the model’s representation capability across different scales and frequency components. Using WaveletPool and WaveletUnPool, the PHF module can efficiently extract and restore multi-scale frequency-domain features, and further optimize weighted feature fusion via AIFI HiLo’s high- and low-frequency attention mechanism. Overall, the PHF module provides a more accurate and efficient solution for small-target detection, especially for recognizing targets with low contrast and small size.

## Experiments and results analysis

### Experimental setup

#### Experimental environment.

The experimental hardware platform consists of an Intel(R) Xeon(R) Platinum 8470Q CPU (2.10 GHz) and an NVIDIA GeForce RTX 4090 GPU with 24 GB of video memory. The software environment includes Ubuntu 20.04, Python 3.8, PyTorch 1.10.0, and CUDA 11.3. No pre-trained weights were used during training. For the RT-DETR model, the batch size was set to 4, the number of training epochs was set to 200, and the input image size was 640 × 640. All other hyperparameters followed the official training configuration.

#### Evaluation metrics.

The evaluation metrics used in this study include precision (P), recall (R), mAP@0.5, mAP@0.5-0.95, the number of parameters, and model complexity measured by FPS (frames per second). Precision is defined as the proportion of true positives (TP) among all samples predicted as positive by the model (TP + FP), reflecting the model’s accuracy in positive predictions. The formula is as follows:


$$\begin{array}{c} \text{Precision}=\frac{\text{TP}}{\text{TP}+\text{FP}} \end{array}$$ (9)

Recall is defined as the proportion of true positives (TP) among all ground-truth positive samples (TP + FN), reflecting the model’s sensitivity to positive samples. The formula is as follows:


$$\begin{array}{c} \text{Recall}=\frac{\text{TP}}{\text{TP}+\text{FN}} \end{array}$$ (10)

FPS denotes the number of frames processed per second, and TP refers to the inference time for a single image frame. The formula is as follows:


$$\begin{array}{c} \text{FPS}=\frac{\text{1}}{{\text{t}}_{\text{p}}} \end{array}$$ (11)

mAP is a comprehensive metric that jointly considers precision and recall, reflecting the overall performance of the model across multiple categories. A higher value indicates better detection performance across different targets. AP (average precision) is the average precision for a single category, and mAP is obtained by averaging the AP values over all categories, providing an evaluation of overall multi-class detection performance. mAP@0.5 is commonly used to measure detection accuracy and is calculated by averaging AP across all categories at an IoU (intersection over union) threshold of 0.5. In contrast, mAP@0.5-0.95 averages AP over multiple IoU thresholds from 0.5 to 0.95, offering a more comprehensive evaluation under varying matching criteria. The formula is as follows:


$$\begin{array}{c} \text{AP}=\int_{\text{0}}^{\text{1}}\text{PdR} \end{array}$$ (12)

$$\begin{array}{c} \text{mAP}=\frac{\sum_{\text{i}=\text{1}}^{\text{N}}\text{A}{\text{P}}_{\text{i}}}{\text{N}} \end{array}$$ (13)

### Experimental dataset

The effectiveness of the proposed model is assessed on the VisDrone2019 dataset, a widely adopted benchmark for aerial object detection. Collected by unmanned aerial vehicles (UAVs), the dataset encompasses images captured across diverse flight altitudes, geographic locations, and temporal conditions, thereby reflecting a broad spectrum of real-world scenarios, including urban and suburban environments, parks, and complex road networks. VisDrone2019 is partitioned into 6,471 training images, 548 validation images, and 1,610 test images, covering ten object categories with a pronounced imbalance in class distribution.

**Fig 5:**
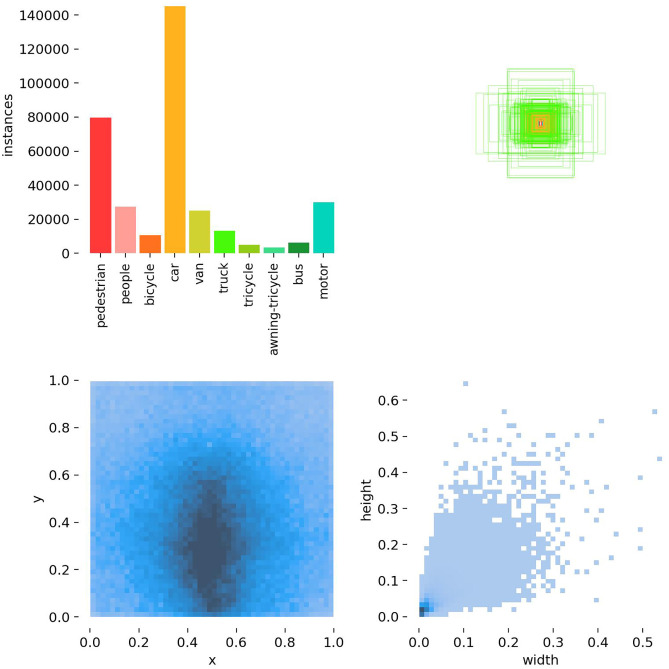
Datasets label distribution map.

Beyond the dataset split, the intrinsic challenges of aerial object detection are further characterized through an analysis of target attribute distributions. As shown in Fig 5, the dataset is overwhelmingly dominated by small-scale objects exhibiting substantial variability in size, with normalized width and height distributions densely concentrated in low-value regions. Moreover, object instances display a clear tendency toward spatial centralization within the image plane. These characteristics—specifically the prevalence of small objects and severe scale imbalance—make fine-grained feature representations highly vulnerable to degradation during down-sampling and cross-scale feature fusion. As a result, VisDrone2019 places stringent demands on the multi-scale feature extraction and fusion capabilities of detection algorithms. Owing to these challenging properties, the dataset constitutes a rigorous and representative benchmark for evaluating aerial small-object detection methods.

### Ablation experiments

To evaluate the contribution of each proposed module to model performance, ablation experiments were conducted on the VisDrone2019 training set. The RT-DETR-R18 model was used as the baseline, and three modules were introduced sequentially: A (Base + Faster-Rep), B (Base + PHF), and C (Base + P2). Additionally, several module-combination experiments (A + B, A + C, B + C, and A + B + C) were conducted to verify potential synergistic effects among the modules. As shown in Table 1, model A adopts the Faster-Rep module as the backbone feature-extraction structure, reducing the number of parameters and computational cost by 15.1% and 12.3%, respectively, while improving mAP@0.5 by 1.6%. This demonstrates the effectiveness of the lightweight design in improving model efficiency. Model B introduces the PHF (Pyramid Hierarchical Frequency-Domain Fusion) module, which strengthens multi-scale feature fusion by combining wavelet pooling with the high- and low-frequency attention mechanism, resulting in a 1.0% increase in mAP@0.5. Model C adds the P2 detection head to better capture shallow features for small targets, leading to a clear improvement in small-target detection; its mAP@0.5 is 3.0% higher than that of the baseline model.

**Table 1:** Ablation experiments.

	Params(M)	GFLOPs	P(%)	R(%)	mAP0.5(%)	mAP0.5–0.95(%)	FPS/(1bs)
Base model	20.18	58.6	61.4	45.8	47.1	28.9	101.2
A	17.14	51.4	61.1	47.2	48.7	30.2	103.4
B	19.39	62.7	60.9	47.1	48.1	29.5	112.9
B1	20.15	58.8	62.0	46.2	47.4	29.1	105.5
C	18.92	81.7	62.6	48.3	50.1	31.2	94.4
A + B	16.25	55.1	63.9	47.1	49.1	30.3	101.2
B + C	18.18	86.4	62.4	48.8	50.9	32.2	94.7
A + C	15.88	74.4	63.1	48.8	50.8	31.7	87.3
A + B + C	15.13	79.1	63.3	50.5	52.5	33.1	86.9

In addition, model B1 (Base + AIFI-HiLo) was introduced to evaluate the impact of the AIFI-HiLo module. The results show that the AIFI-HiLo module improves mAP@0.5 by 0.3% over the baseline, demonstrating its ability to enhance detection accuracy for small objects while maintaining reasonable computational efficiency.

In the combination experiments, the A + B model integrates backbone optimization with the frequency-domain feature enhancement module, further improving overall performance compared with using either module alone. The A + C model preserves shallow-level details while maintaining a lightweight design, achieving better detection performance. The B + C model combines high- and low-frequency feature fusion with a multi-level detection structure, and the mAP@0.5-0.95 reaches 32.2%. Finally, the complete model A + B + C (PF-DETR) integrates all three optimization strategies. While reducing the number of parameters by 25.0%, PF-DETR achieves 63.3% and 50.5% in precision (P) and recall (R), respectively, which are 1.9% and 4.7% higher than the baseline. The mAP@0.5 reaches 52.5%, and mAP@0.5-0.95 increases by 1.0%, showing a clear improvement over the original RT-DETR-R18 model and demonstrating the effectiveness and robustness of the proposed method.

**Fig 6:**
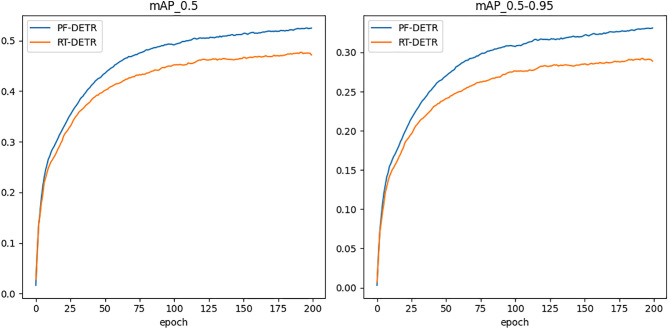
Comparison of Training Curves.

Fig 6 visually compares the training curves of the improved PF-DETR model and the baseline RT-DETR-R18 model in terms of mAP@0.5 and mAP@0.5-0.95. As shown, PF-DETR consistently outperforms the baseline on both metrics and exhibits relatively stable and faster convergence.

In summary, PF-DETR achieves significantly higher detection accuracy, demonstrating the effectiveness of the proposed improvements.

### Comparative experiments

To further validate the effectiveness of the proposed algorithm, we evaluate it on the VisDrone2019 validation set under the same training settings and compare it with several representative detectors, including the YOLO series and other representative methods for small-object detection. We report AP, mAP, the number of parameters (Params), and inference speed measured by frames per second (FPS) to jointly evaluate detection accuracy, model complexity, and computational efficiency. The comparative results are summarized in Table 2.

**Table 2:** Comparative results of different algorithms in average precision and parameters on VisDrone dataset.

Algorithms	AP/%										mAP/%	Params(M)	FPS/(1bs)
	pedestrian	people	bicycle	car	van	truck	tricycle	awning-tricycle	bus	motor			
Faster R-CNN	20.9	14.8	7.3	51.0	29.7	19.5	14.0	8.8	30.5	21.2	21.8	45.70	20.6
YOLOv5s	38.9	31.2	10.9	73.4	35.2	29.3	20.3	10.7	42.9	36.8	33.0	7.03	165.4
TPH-YOLOv5	53.2	42.1	21.3	83.4	45.1	42.4	33.1	16.4	61.1	51.2	44.9	60.33	33.3
YOLOv8n	34.0	26.4	7.21	75.3	38.4	28.1	20.8	11.0	45.6	34.8	32.2	3.20	232.9
YOLOv8s	41.8	32.6	12.4	79.2	44.5	35.3	26.5	17.1	54.0	43.3	38.7	11.13	167.5
YOLOv8l	50.9	39.7	21.8	82.3	49.2	**45.0**	37.7	20.2	66.9	52.1	46.6	43.67	116.7
YOLOv11n	31.2	25.3	8.0	74.6	37.7	26.3	19.2	11.5	43.3	34.3	31.1	2.57	268.8
YOLOv11s	41.5	31.4	11.3	79.4	44.8	35.3	26.1	15.2	55.1	43.1	38.3	9.46	201.4
RT-DETR	55.3	49.3	21.4	85.6	51.3	40.1	33.6	18.8	61.8	59.6	47.7	19.88	101.2
PF-DETR	61.0	52.6	26.9	87.7	56.8	43.4	39.0	21.3	71.6	64.4	52.5	14.76	86.9

As shown in Table 2, PF-DETR achieves superior performance in most categories, particularly for small objects such as pedestrian, bicycle, and tricycle, and is only slightly inferior to YOLOv8l in the truck category (43.4% vs. 45.0%). Meanwhile, PF-DETR contains only 14.76M parameters, which is significantly fewer than some larger high-accuracy models. In terms of inference efficiency, PF-DETR achieves 86.9 FPS, demonstrating competitive real-time performance while maintaining higher detection accuracy. Although lightweight models such as YOLOv11n and YOLOv8n use fewer parameters (2.57M and 3.2M, respectively), their mAP values (31.1% and 32.2%) are considerably lower. Overall, these results indicate that PF-DETR achieves a favorable balance between detection accuracy, model size, and inference efficiency for UAV aerial object detection.

To further assess the effectiveness of the proposed method, additional comparative experiments were conducted on the VisDrone2019 test set. We compare PF-DETR with detectors of different computational complexities, including YOLOv5m, YOLOv8m, and YOLOv10m, as well as recently published Transformer-based detection frameworks designed for UAV aerial imagery, such as UAV-DETR [28] and ACD-DETR [15]. All methods are evaluated on the same task and dataset to ensure a fair and consistent comparison. For methods with publicly available implementations, the official source code and released weights provided by the authors were used to reproduce the results under the same experimental environment whenever possible.

The quantitative results are summarized in Table 3. Compared with YOLO detectors of different computational scales, PF-DETR achieves an mAP@0.5 of 41.5%, surpassing YOLOv5m, YOLOv8m, and YOLOv10m by 6.6%, 6.2%, and 7.7%, respectively. Meanwhile, PF-DETR maintains a relatively compact model size with only 14.76M parameters, which is smaller than several detectors with comparable performance.

**Table 3:** Comparison experiment.

Algorithms	Params(M)	GFLOPs	mAP0.5(%)	mAP0.5–0.95(%)	P(%)	R(%)	Latency (ms/img)
YOLOv5m	20.88	48.0	34.9	20.1	47.7	36.8	—
YOLOv8m	25.85	78.7	35.3	20.3	47.9	37.6	—
YOLOv10m	15.46	59.1	33.8	19.4	47.6	35.2	—
UAV-DETR-EV2	13.01	42.9	38.1	22.2	55.5	39.1	15.1
UAV-DETR-R18	21.26	72.5	41.4	24.4	57.0	41.8	23.8
ACD-DETR	16.30	68.9	39.4	22.8	56.3	41.0	32.0
ACD-DETR-SBA+	15.67	94.2	41.5	23.8	57.2	42.2	17.1
RT-DETR-R18	19.88	57.0	37.7	21.7	54.5	39.3	8.2
PF-DETR	14.76	75.5	41.5	24.5	57.3	42.2	10.7

Note: Latency denotes the actual inference latency measured on an NVIDIA GeForce RTX 4090 with batch size 1 and an input size of 640 × 640.

In addition, when compared with recently proposed UAV-oriented detection frameworks such as UAV-DETR-R18 and ACD-DETR, PF-DETR achieves comparable or slightly better detection accuracy while maintaining favorable model efficiency. In terms of practical deployment efficiency, PF-DETR achieved an actual inference latency of 10.7 ms/img on an NVIDIA GeForce RTX 4090 with batch size 1, compared with 8.2 ms/img for RT-DETR-R18. This result indicates that the improved detection accuracy is accompanied by an acceptable increase in real-time inference latency. These results demonstrate that the proposed PF-DETR framework achieves competitive performance on the VisDrone2019 benchmark for UAV small-object detection. CGMDet [29] and Pc-DETR [30] were also considered for inclusion in Table 3; however, no official publicly available implementation could be verified at the time of revision, and these methods therefore could not be reliably reproduced under the same experimental setting.

### Generalization experiments

To further evaluate the effectiveness and generalization capability of the PF-DETR algorithm, this section presents generalization experiments on the RUOD dataset and the TinyPerson dataset. The quantitative results of the generalization experiments on the TinyPerson and RUOD datasets are summarized in Table 4.

**Table 4:** Generalization performance across diverse scenarios.

Algorithms	TinyPerson			RUOD		
	mAP0.5(%)	P(%)	R(%)	mAP0.5(%)	P(%)	R(%)
RT-DETR-R18	21.1	41.7	24.9	83.2	83.9	78.8
PF-DETR	23.1	42.0	27.7	85.6	84.9	79.2

(1) The TinyPerson dataset [31] focuses on small-target detection and contains 1,610 images. Of these, 794 images, primarily from open beach scenes, are selected as the training set. The dataset emphasizes detecting people in aerial and long-range imagery, with target instances generally sparse, although local clusters may occur in some areas, requiring the model to provide accurate localization. Additionally, complex backgrounds such as beaches, water surfaces, and occlusions increase the difficulty of distinguishing small targets from the background, making the detection task more challenging.
(2) The RUOD dataset [32] targets small-object detection in diverse underwater scenes. It contains 14,000 images, of which 9,800 are randomly selected for training and 4,200 for testing. The dataset includes 10 categories, such as sea urchins and sea cucumbers. A key challenge is the color shift caused by light attenuation and scattering underwater, which often leads to blurred targets that are difficult to distinguish. This dataset, therefore, provides a comprehensive benchmark for evaluating the robustness and effectiveness of detection algorithms in complex environments.

The generalization experiments train the models on the training dataset and evaluate them across distinct datasets. PF-DETR shows superior overall performance to RT-DETR-R18 in cross-dataset evaluations. Notably, under complex conditions involving small object detection, PF-DETR exhibits enhanced robustness and accuracy. On the TinyPerson dataset, PF-DETR achieves substantial gains in both precision and recall, with particularly pronounced improvements for low-contrast and small-scale targets. Consistent trends are observed on the RUOD dataset, where PF-DETR attains higher mAP and recall across diverse challenging scenarios. Together, these results suggest that the proposed improvements in PF-DETR are not specific to a particular dataset but instead reflect enhanced multi-scale feature extraction and fusion capabilities that generalize effectively to complex small object detection tasks with heterogeneous characteristics.

### Visualization analysis

**Fig 7:**
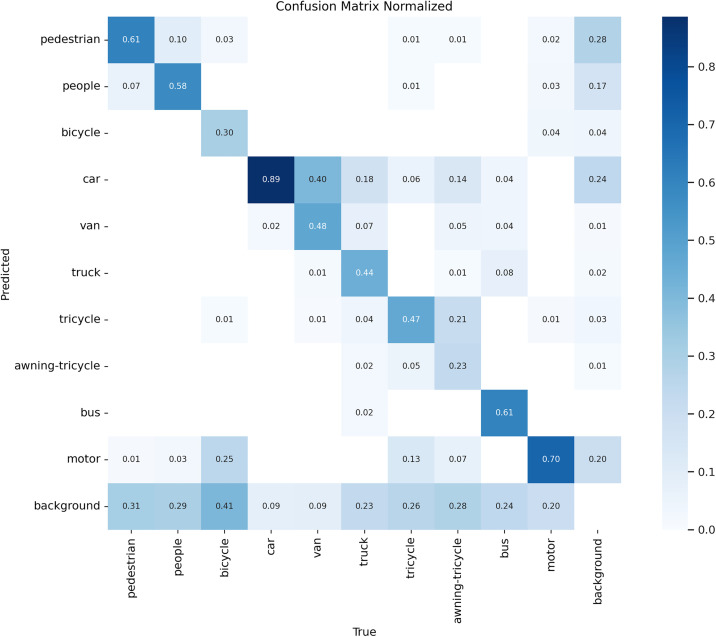
Confusion matrix of RT-DETR-R18.

To more intuitively demonstrate the advantages of the improved PF-DETR model for small-target detection in complex scenes, Fig 7 shows the confusion matrix of RT-DETR-R18 on the VisDrone2019 dataset. As can be observed, several small-object categories still suffer from relatively low diagonal values, indicating weaker classification accuracy and less stable recall in cluttered aerial backgrounds.

**Fig 8:**
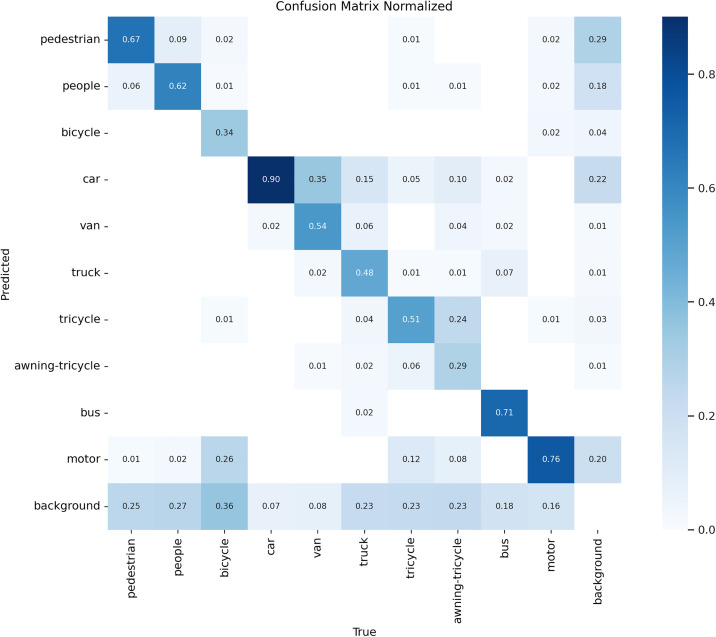
Confusion matrix of PF-DETR.

In contrast, Fig 8 presents the confusion matrix of PF-DETR on the same dataset. Compared with RT-DETR-R18, PF-DETR shows overall higher diagonal values, indicating more accurate classification and more stable recall across categories, with particularly notable gains for small objects such as pedestrian, people, bicycle, bus, and motor. Meanwhile, the values in the bottom row (background) are generally lower, suggesting that PF-DETR reduces the tendency to misclassify objects as background and is therefore less affected by background noise. In addition, PF-DETR alleviates confusion between visually similar classes (e.g., tricycle vs. awning-tricycle) as well as among vehicle categories, demonstrating improved class discrimination in complex aerial scenarios.

**Fig 9:**
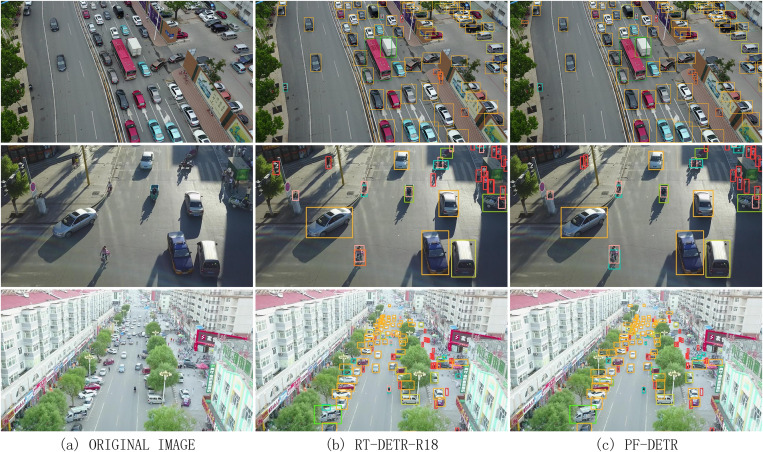
Visualization of Detection in Different Scenes of the VisDrone2019 Dataset.

To further illustrate qualitative detection differences, Fig 9 presents the detection results of RT-DETR and PF-DETR on the VisDrone2019 dataset. It can be observed that RT-DETR suffers from missed detections and false positives for small targets under varying lighting conditions and significant changes in target density, such as misidentifying shadows as pedestrians. Additionally, since small targets occupy only a limited number of pixels, the model also misclassifies tree pits as motorcycles and street lamps as pedestrians.

The improved PF-DETR model effectively detects small targets that are missed by the baseline model. When targets are occluded, the improved model better captures their overall characteristics and maintains higher confidence for occluded instances. In particular, in urban intersection scenes with dense car, van, and truck instances and severe occlusion, the baseline RT-DETR-R18 model is prone to missed detections and inaccurate bounding boxes for overlapping targets. In contrast, PF-DETR maintains a higher recall under the same conditions and distinguishes adjacent targets more accurately. These visualizations clearly demonstrate the advantages of PF-DETR in challenging scenarios involving dense targets, small targets, and occluded targets.

**Fig 10:**
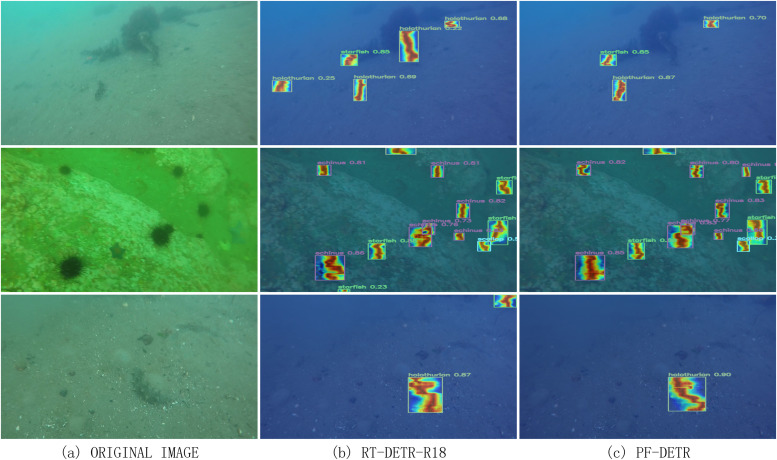
Visualization of Detection in Different Scenes of the RUOD Dataset.

A heatmap is a visualization technique widely used in object detection to intuitively illustrate the model’s attention to different regions of an image. The color intensity reflects the activation level of the corresponding regions, with highlighted areas indicating where the model focuses most. To further evaluate the detection capability of the improved model for slender, occluded, and blurred targets, Fig 10 presents the detection results of RT-DETR and PF-DETR on the RUOD dataset. It can be seen that PF-DETR achieves markedly better detection performance on the RUOD dataset than RT-DETR.

In particular, for small targets in underwater environments, PF-DETR can localize and detect slender, blurred, and partially occluded targets more accurately. As shown in the figure, for sea urchins with indistinct boundaries and reefs with shapes similar to sea cucumbers, PF-DETR focuses more effectively on the main target region than the baseline model. In contrast, RT-DETR often produces missed detections or false recognitions in these cases. The heatmap in the figure clearly illustrates the model’s attention across different regions. PF-DETR shows more concentrated and accurate attention on the target, especially in scenes with complex backgrounds and low contrast, where the high-activation regions enable better identification of small targets.

These results indicate that PF-DETR has clear advantages in complex underwater environments and can better handle diverse challenges such as blurred targets, complex backgrounds, and target occlusion.

## Conclusion

Based on RT-DETR, this paper proposes an enhanced detection framework, PF-DETR, for small-target detection in complex scenes. By integrating the structural designs of FasterNet and RepVGG into the backbone, a lightweight FasterNet_Rep module is constructed to effectively reduce computational complexity and parameter count. A Pyramid Hierarchical Frequency-Domain Fusion (PHF) module is also designed by combining WaveletPool with the AIFI-HiLo high- and low-frequency attention mechanism, enabling efficient multi-scale feature fusion and enhanced detail representation. Additionally, a P2 detection head is introduced to strengthen the perception of shallow features for small targets.

Experimental results on the VisDrone2019 dataset show that PF-DETR maintains a high detection speed while improving mAP@0.5 by 5.4% and reducing the number of parameters by 25.0%, achieving a practical balance between accuracy and lightweight design. PF-DETR also demonstrates strong generalization and robustness on the TinyPerson and RUOD datasets, further validating its effectiveness for small-target detection in complex UAV scenes.

Future work will further explore lightweight design and energy consumption optimization, as well as extend the application potential of PF-DETR to complex scenarios such as defect detection in photovoltaic power generation and multi-source information fusion.
